# Corneal Refractive Procedures for the Treatment of Presbyopia

**DOI:** 10.2174/1874364101711010059

**Published:** 2017-04-27

**Authors:** Kareem Moussa, Naz Jehangir, Tova Mannis, Wai L. Wong, Majid Moshirfar

**Affiliations:** 1 Department of Ophthalmology, Francis I. Proctor Foundation, University of California San Francisco, San Francisco, CA 94143, USA; 2 Research Associate, Francis I. Proctor Foundation, University of California San Francisco, San Francisco, CA 94143, USA; 3 Clinical fellow, Francis I. Proctor Foundation, University of California San Francisco, San Francisco, CA 94143, USA; 4 Medical Director HDR Research Center, Hoopes Vision and Professor of Ophthalmology, Department of Ophthalmology and Visual Sciences, John A Moran Eye Center, University of Utah School of Medicine, Salt Lake City, UT, USA

**Keywords:** Presbyopia, LASIK, PresbyLASIK, Corneal Inlays, Conductive Keratoplasty, INTRACOR

## Abstract

**Purpose:**

Refractive surgery has been in use for a long time and is evolving at a fast pace with several new corneal procedures being used for the correction of presbyopia. The purpose of this article is to give a comprehensive review of the literature to evaluate the outcome and success of different corneal refractive surgical procedures in presbyopic patients.

**Methods:**

We performed a comprehensive search on PubMed to identify published reports of the various procedures utilized in the past and present to correct presbyopia. The outcomes of these procedures were recorded.

**Results and conclusion:**

We found that varying rates of success have been reported with these procedures. The results of our exhaustive search are presented in this report for review.

## INTRODUCTION

Presbyopia is the inevitable loss of lens accommodative power that is usually first noticed between the ages of 40 and 50. There are two main mechanisms that have been proposed to explain the physiologic changes that lead to presbyopia. According to the Helhmholtz theory, ciliary mucle contraction results in decreased zonular tension, permitting the lens to take a more globular shape and increase its power [1]. As the lens ages, it loses its elastic properties and is unable to take on globular shape in response to ciliary muscle contraction, leading to loss of accommodation. The Schachar theory suggests that contraction of the longitudinal ciliary muscle results in reduced tension and subsequent relaxation of the anterior and posterior zonules, and increased tension on the equatorial zonules, which increases the power of the lens due to a decrease in the peripheral volume of the lens and an increase in the central volume of the lens [2]. As the lens ages, the equatorial diameter increases and the peripheral volume increases, leading to an eventual loss of accommodative power. The age at which presbyopia becomes clinically significant is dependent on a host of factors, including accommodative ability, distance refraction, age, sex, ethnicity, and geographic location [3-10]. 

In 2005, it was estimated that 1.04 billion people suffer from presbyopia, 67% of whom live in the developing world [11]. Among individuals with presbyopia, 517 million do not have adequate correction. Studies report significant functional impairment as a direct result of presbyopia, particularly in the developing world; using their habitual spectacles, 70% of rural Tanzanians, 58% of Brazilians, and 53% of Indians report difficulty with near tasks. In the United States, a multicenter study reported an association between presbyopia and substantial negative effects on quality of life [12]. Worldwide, it is estimated that the presbyopic population will increase to 1.4 billion by 2020 and 1.8 billion by 2050, with uncorrected presbyopia afflicting 563 million people by 2020.

Spectacles are widely used to correct presbyopia. The high levels of patient satisfaction achieved with various refractive procedures to correct refractive errors have led to an interest in and the development of a number of surgical techniques to correct presbyopia and minimize or eliminate the need for spectacle wear. In this review, we specifically discuss corneal surgical techniques for the treatment of presbyopia.

## MATERIALS AND METHODS

Using PubMed, an extensive literature search was performed to identify studies published from 1964 to May 2015 that evaluated outcomes of the various procedures described in this paper. We searched for articles in English and French. Keywords included presbyopia, accommodation, refractive surgery, LASIK, PRK, corneal inlays, Kamra inlay, Flexivue inlay, Raindrop inlay, conductive keratoplasty and IntraCor. The safety, efficacy and predictability of these refractive procedures are summarized in tables and graphs.

### LASIK and PRK Monovision

Monovision is an attempt to provide acceptable distance and near vision that eliminates the need for spectacle wear by optimizing one eye for near vision, typically the non-dominant eye, and optimizing the other eye, typically the dominant eye, for distance vision. This is known as conventional monovision. Alternatively, optimizing the non-dominant eye for monovision is termed crossed monovision. Contact lens wear has been used to achieve monovision with a success rate of 76% [13].

Refractive surgery to induce permanent monovision has been extensively studied. In 2001, Jain *et al.* analyzed outcomes of 42 patients who had undergone photorefractive keratectomy (PRK), photoablative astigmatic refractive keratectomy (PARK), or laser in situ keratomileusis and PARK (LASIK-PARK) in one or both eyes with the goal of achieving conventional monovision, in which the non-dominant eye is corrected for near vision, or crossed monovision, in which the dominant eye is corrected for near vision [14]. They reported a satisfaction rate of 88%, suggesting that patient satisfaction after refractive surgery monovision is higher than that after contact lens monovision. Other studies have reported similar satisfaction rates, ranging from 85-98% (Table **1**). While this is an encouraging outcome in the quest to eliminate spectacle or contact lens dependence, other studies suggested worse outcomes following LASIK with increasing age [15-21]. In 2007, Ghanem *et al.* evaluated LASIK outcomes in 511 myopic eyes and 199 hyperopic eyes subdivided into three groups of varying age (40-49 years old, 50-59 years old, and 60-69 years old) [22]. They found that while older patients had a trend toward worse final best spectacle-corrected visual acuity (BSCVA) and higher retreatment rates, these differences were not statistically significant, suggesting LASIK is a reasonably safe and efficacious procedure in the presbyopic age group.

While monovision has proven to be a successful method of treating presbyopia, it does carry the risk of inducing anisometropia and reducing binocular visual acuity and stereopsis, which should be discussed pre-operatively [13].

### PresbyLASIK

Accommodation is a dynamic, active process that is dependent on the elastic properties of the lens. The cornea, in comparison, is a static tissue that does not alter its shape to influence refractive power. Relative to the elastic lens, it has a low depth of field. Naturally, alterations in the cornea to enhance its depth of field may serve as a potential solution for presbyopia. This has led to recent interest in the development of a multifocal cornea via laser ablation for this purpose. LASIK has garnered more attention as the procedure of choice for corneal multifocality, as it avoids the potential problem of suboptimal epithelium growth over the ablated areas [23].The various techniques to create a multifocal cornea have been named presbyLASIK techniques, of which there are three: transitional multifocality, peripheral presbyLASIK, and central presbyLASIK. The creation of a transitional multifocal cornea has been associated with significant vertical coma and has limited the popularity of this technique [23].

In peripheral presbyLASIK, peripheral cornea is ablated for near (Fig. (**1**), b= near vision) and the center is left for distance (Fig. (**1**), a= distance vision). In myopes undergoing peripheral presbyLasik, a significant amount of corneal tissue must be removed to create a hyper-negative ablation profile. Thus, peripheral presbyLASIK is typically performed in presbyopic hyperopes or presbyopic low myopes. In 2009, Uy *et al.* evaluated outcomes of presbyLASIK in 195 eyes with myopic presbyopia and 119 eyes with emmetropic or hyperopic presbyopia. 83% of the myopic presbyopes achieved both 20/30 or better uncorrected distance visual acuity (UDVA) and J3 or better uncorrected near visual acuity (UNVA) at three-month follow-up [24]. 87% of the emmetropic or hyperopic presbyopes achieved the same outcome. In a study evaluating monocular presbyLASIK in the non-dominant eye and monofocal refraction-based LASIK in the dominant eye in 75 presbyopic myopes and 28 presbyopic hyperopes, Epstein *et al.* reported complete spectacle independence in 92% of the myopes and 89% of the hyperopes at mean follow-up of 27.4 months [25]. These findings lend support to the use of peripheral presbyLASIK for presbyopic correction, particularly in hyperopes and low myopes.

In central presbyLASIK, the center of the cornea is corrected for near (Fig. (**1**), a= near vision) while the peripheral cornea (Fig. (**1**), b= distance vision) is left for distance. This technique is associated with only minimal corneal excision, thus it is suitable for both myopes and hyperopes. A study by Alio *et al.* in 2006 evaluated central presbyLasik in a hyperopic population and found that 64% of patients achieved UDVA of 20/20 or better, and 72% achieved UNVA of 20/40 or better at 6-month follow-up; however, 20% lost two lines of BSCVA, contrast sensitivity was reduced, the coefficients for coma increased, and the coefficients for spherical aberrations decreased [26]. In 2008, Jung *et al.* reported UDVA of 0.8 (20/25) or better and UNVA of 0.65 (J2 approx.) or better in 64.3% (9/14 patients) of hyperopic presbyopes who received central presbyLASIK treatment; no significant changes were found in contrast sensitivity or total higher order aberrations [27]. Another study of 26 eyes reported an improvement in mean UNVA from 0.15 (J11 approx.) to 0.68 (J2 approx.) following central PresbyLASIK; mean UDVA improved from 0.35 (20/60 approx.) to 0.8 (20/25) [28].

These studies validate central presbyLASIK as a technique to improve functional near vision, however the possibility of reduced quality of vision does exist. Satisfaction rates of presbyLASIK have ranged from 76% - 100% (Table **2**).

### Corneal Inlays

In 1964, Barraquer introduced keratophakia, a procedure in which a lamellar incision is made through the corneal stroma, and a lenticule is placed to augment the cornea’s refractive power, as a treatment for hyperopia and presbyopia [29]. While this procedure resulted in unpredictable results and has been largely abandoned, it led to the development of other intra-corneal inlays, some of which have also been met with unfortunate outcomes, including corneal necrosis and opacification [30-33]. In this article, we will be discussing the following four inlay designs.

#### 1 Kamra Inlay

**Fig. (2):**
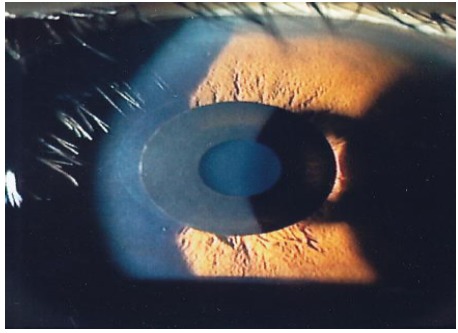
Kamra inlay. Permission for use of this image was obtained from AcuFocus, Inc. Photocredits Dr Minoru Tomita.

The Kamra inlay is a corneal inlay manufactured by AcuFocus Inc., in Irvine, California, for the treatment of presbyopia (Fig. **2**). It is likely the most well-studied corneal inlay and as of April 2015, has earned approval from the U.S. Food and Drug Administration (FDA) for the surgical treatment of presbyopia in the U.S patients 45-60 years old who do not require distance correction, have not undergone cataract surgery, and require a near correction of +1.00 to +2.50 diopters. The Kamra corneal inlay, model ACI7000 in the studies mentioned below, is 10 uM thick, composed of polyvinylidene fluoride, with an outer diameter of 3.8 mm and a central aperture of 1.6 mm. The inlay has 1600 micro-perforations, each 25 uM in diameter, allowing nutrient flow through the cornea. The central aperture allows for an increased depth of focus to improve near and intermediate visual acuity, with minimal effects on distance visual acuity. The model of the Kamra inlay that is currently available is ACI7000PDT. It is thinner than the previous model, with a thickness of 5 uM, and has 8400 smaller micro-perforations ranging in size from 5 to 11 uM, which allow an average light transmission of 6.7%, a decrease from 7.1%; these changes are thought to reduce some of the visual symptoms experienced with the inlay, such as glare.

A study evaluating the long-term outcomes of monocular Kamra implantation in emmetropic presbyopes reported an improvement in mean binocular UNVA from J6 to J2 and an improvement in mean uncorrected intermediate visual acuity (UIVA) from 20/32 to 20/25 at 60 months of follow-up [34]. Mean UDVA decreased slightly from 20/12.5 to 20/16. 74.2% of patients had UNVA of J3 or better, 87.1% had UIVA or 20/32 or better, and 93.5% had UDVA of 20/20 or better. Out of 32 inlays, one was removed due to patient dissatisfaction from a hyperopic shift in the operative eye; there was no loss of CDVA or CNVA 2 years after the removal. In a similar study that evaluated the long-term visual results of the Acufocus ACl7000 (now Kamra) intra-corneal inlay in 39 presbyopic emmetropic (naturally or post-LASIK) phakic patients, all 22 patients who presented for the 4-year follow-up visit gained at least 2 lines in UNVA with no significant loss in distance vision, with a final mean UNVA of J1 in the operative eye [35]. The mean gain in UNVA was 3.8 lines. The UDVA was 20/40 or better in all eyes at the 4-year follow-up visit, with a slight, statistically insignificant decrease from a mean UDVA of 20/20 pre-operatively to a mean UDVA of 20/25 postoperatively. Four inlays underwent explantation: one at six weeks due to a buttonhole flap, two due to refractive shifts (one myopic, -2.00D, and one hyperopic, +3.00D) with significant glare and halos, and one due to a thin flap of 58 uM that was not noticed during surgery. All four eyes that underwent explantation returned to within ±1.00D of the preoperative refraction.

Other studies have reported encouraging results of Kamra implantation following Lasik surgery as well as in combination with Lasik surgery, with highest patient satisfaction reported in older patients; occasional postoperative symptoms include dry eyes, halo, and glare [36-38].

#### 2 Raindrop Inlay

**Fig. (3):**
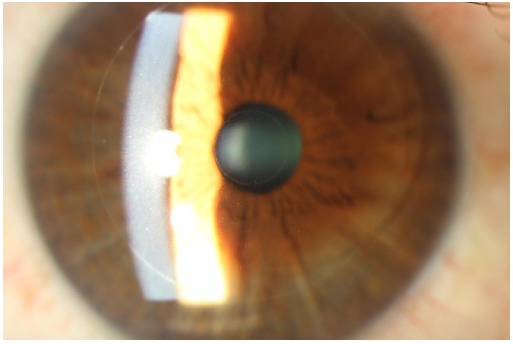
Raindrop inlay, courtesy of Pf. Choun-Ki Joo, MD, PhD.JPG.

The Raindrop near vision inlay is a corneal inlay manufactured by Revision Optics in Lake Forest, California, that aims to alter the shape of the cornea to modify its refractive power. It has a 2.0 mm diameter with a central thickness of 32 to 36 uM (Fig. **3**). It is made of permeable hydrogel material that has the same refractive index as the cornea, but is thicker in the center and thinner at the edges. It is placed in the stromal bed underneath a keratotomy flap. A study of 20 emmetropic presbyopes who underwent monocular implantation of the Raindrop inlay in the non-dominant eye reported at least 20/40 UNVA in all eyes that underwent treatment at one-year follow-up [39]. There was minimal effect on UDVA, however one patient was dissatisfied with the resulting vision and underwent explantation of the inlay. Simultaneous monocular hydrogel inlay implantation in the non-dominant eye and bilateral Lasik treatment in hyperopic presbyopes also yielded positive results, with all eyes with the inlay achieving UNVA of 20/32 or better at one-year follow-up [40]. Mean binocular UDVA improved from 20/53 preoperatively to 20/19 postoperatively. Out of 16 inlays, one was explanted due to recurrent haze. Of the 14 patients analyzed at one-year follow-up, all reported being satisfied or very satisfied with near, distance, and overall vision.

#### 3 Flexivue Inlay

**Fig. (4):**
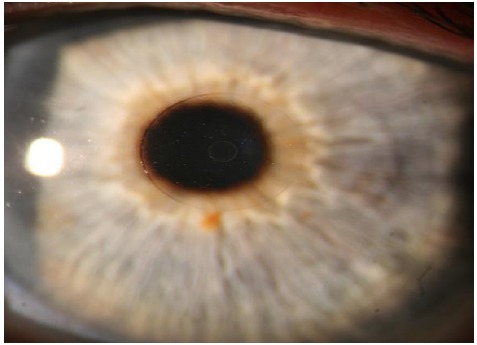
Flexivue Lens. Permission for use of this image was obtained from Presbia Cooperatief, with courtesy Dr Crewe Brown.

**Fig. (5):**
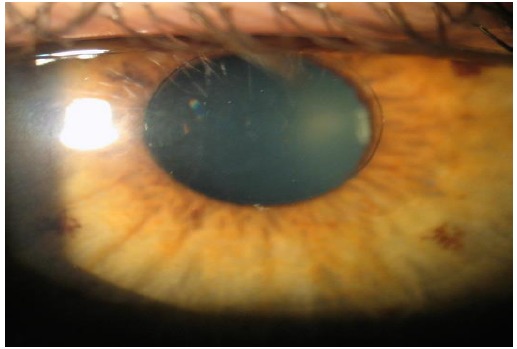
Flexivue Lens. Permission for use of this image was obtained from Presbia Cooperatief, with courtesy Dr Prof Ioannis Pallikaris, Greece.

The Flexivue Microlens is a 3.0 mm diameter corneal inlay with a thickness of 15 uM, manufactured by Presbia Cooperatief in Amsterdam, Netherlands (Figs. **4** and **5**). In a study of 47 emmetropic presbyopes who received the inlay in the non-dominant eye underneath a femtosecond laser flap, UNVA was 20/32 or better in 75% of operative eyes and UDVA decreased significantly from a mean of 20/20 to 20/50 at one-year follow-up [41]. 36% (17/47) of patients lost one line of CDVA in the operated eye, and there was an increase in higher order aberrations as well as a decrease in contrast sensitivity in the operated eye.

#### 4 Icolens Inlay

The Icolens (Neoptics AG) is a 3.0mm corneal inlay with a 1.8mm central zone for distance and a 1.2mm peripheral positive refractive zone for near. There is a 150uM central hole for nutrient flow. The inlay is composed of 2-hydroxyethyl methacrylate and methyl methacrylate, which have hydrogel properties. The power of the refractive zone is dependent on the patient’s near and distance visual acuities, refraction, pupil size and central corneal thickness. Results of a recent study evaluating its efficacy and safety are summarized in Table **3** along with the results of other studies of corneal inlays.

Overall, the use of corneal inlays to treat presbyopia has been met with positive results, however patients should be aware of the possible development of post-operative symptoms such as glare, halos and dry eyes, as well the possibility of decreased distance visual acuity. Despite these risks, patient satisfaction following inlay implantation is high and the demand for this procedure in the U.S is likely to increase following recent U.S FDA approval of the Kamra inlay.

### Conductive Keratoplasty

Conductive keratoplasty (CK) is a non-ablative technique that uses radiofrequency energy to reshape the cornea, approved by the U.S FDA in 2002 for the treatment of previously untreated, low levels of spherical hyperopia (+0.75 to +3.00 diopters). A series of 8 to 32 spots are applied in up to three rings in the peripheral cornea stroma, resulting in shrinking of collagen between the spots and subsequent steepening of the central cornea. It is similar in concept to laser thermal keratoplasty (LTK), which uses a holmium:YAG laser to mechanically steepen the cornea. While LTK gained significant attention in the 1990s, long-term studies showed suboptimal stability of effect [42]. It was hypothesized that the high amount of regression following LTK was due partly to its suboptimal penetration in corneal tissue. Approximately 350 uM of water, which accounts for 75% of the cornea’s stromal mass, is penetrated by the holmium:YAG laser [43]. Based on a corneal thickness of 540 uM, this leads to approximately 65% penetration of corneal depth. CK, on the other hand, penetrates approximately 80% of the cornea’s depth based on histology studies [44].

While CK has proven to be an effective procedure for the treatment of hyperopia, its effects are not permanent. Studies have shown a significant amount of regression in long-term follow-up [45, 46]. One study reported a change in the manifest spherical equivalent from +1.45D pre-operatively, to +0.295D at the 23-month follow-up visit, to +1.394D at mean final follow-up of 73 months; no eye lost more than 1 line of BSCVA [47].

Naturally, the use of CK as an effective, safe, although temporary, treatment for hyperopia led to the investigation of its use as a treatment for presbyopia. A prospective, multicenter, FDA clinical phase III trial evaluating CK’s safety and efficacy in both emmetropic and hyperopic presbyopes found that 66% of the eyes treated for near had a manifest refractive spherical equivalent (MRSE) within 0.5D of the intended correction, 89% were within 1.0D, and 100% were within 2.0D at the six-month follow-up visit [48]. The MRSE mean change was 0.04D per month between months 1 and 3, and 0.06D per month between months 3 and 6. 76% of patients reported being satisfied or very satisfied with the procedure. The results of this study as well as others on the use of CK in the treatment of presbyopia are summarized in Table **4**. In 2004, the U.S FDA granted approval for the use of CK in the treatment of presbyopia in non-dominant eyes with a goal target manifest refraction of -1.00 to -2.00D. While CK has a role in the treatment of presbyopia, the long-term stability of its effects in presbyopic eyes is uncertain. Given the well-documented regression of effect in hyperopic eyes treated with CK, it is likely that a similar effect is observed in presbyopic eyes. CK likely affords an efficacious yet temporary solution to presbyopia.

## INTRACOR

**Fig. (6):**
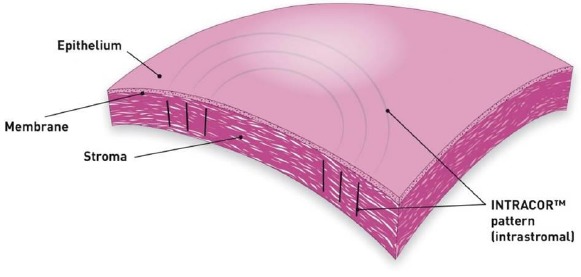
INTRACOR. Permission for use of this image was obtained from Bausch + Lomb Technolas.

INTRACOR is a recently developed procedure that delivers femtosecond laser energy entirely within the stroma to treat presbyopia and low levels of hyperopia. It utilizes the TECHNOLAS femtosecond laser system manufactured by Technolas Perfect Vision GmbH in Munich, Germany. This system delivers laser pulses in a customizable pattern from the posterior stroma to the anterior stroma without impacting the endothelium or Bowman’s layer. This leads to central steepening of the cornea (Fig. **6**). In 2009 Ruiz *et al.* reported an improvement in mean UNVA from 0.27 (20/80 approx.) pre-operatively to 1.0 (20/20) in presbyopic eyes with low amounts of hyperopia or myopia at the 12-month follow-up visit; however, only 27% of eyes were followed for 12 months [49]. UDVA also improved, from 0.86 (20/25 approx.) to 1.07 (20/20 approx.). A mild myopic shift was observed in these eyes with a change in mean spherical equivalent refraction from +0.23 to -0.30. 2.4% of eyes lost 2 lines of CDVA at the 6-month follow-up; these eyes were not followed for 12 months so it is unknown if the lost lines were regained. Of the eyes that were followed for 12 months, none lost any lines. In 2011, Holzer *et al.* reported similar results in presbyopic eyes with mild hyperopia that underwent treatment with INTRACOR, with much better retention of patients (92%) at the 12-month follow-up visit [50]. Mean UNVA improved from 0.7 logMAR (20/100) pre-operatively to 0.2 logMAR (20/32). Mean UDVA remained stable at 0.1 logMAR (20/25). A median myopic shift of 0.5D was observed. 7.1% of patients had lost 2 lines of CDVA at the 12-month follow-up visit. 71.4% of patients were satisfied with the procedure. Based on a subjective questionnaire, glare and halos were felt to be mildly noticeable.

A recently published study by Khoramnia *et al.* evaluated a modified delivery of the laser pulses over six concentric rings instead of the standard five to test the assumption that a larger optical zone would result in even greater improvement of near visual acuity [51]. As expected, a greater improvement in UNVA was observed over a 36-month follow-up period, however a greater loss of CDVA was also observed compared to previously reported loss of CDVA in prior studies, leading the authors to recommend the standard five-ring laser pulse delivery technique.

The above studies demonstrate INTRACOR’s efficacy in the treatment of presbyopia with substantial improvements in UNVA with minimal effect on UDVA. Photopic side effects seem to be minimal, however the loss of CDVA does raise concerns regarding the safety of the procedure, particularly given its irreversible nature. In 2013, a case report was published describing the development of unilateral keratectasia in a patient who underwent unilateral INTRACOR treatment for presbyopia, further questioning the safety of this procedure [52]. While many patients have fared well with INTRACOR, its effects on corneal mechanical stability are uncertain at this time and merit further study. Table **5** summarizes the results of recent studies on INTRACOR as a treatment option for presbyopia.

### Collective Data Analysis

**Graph (1):**
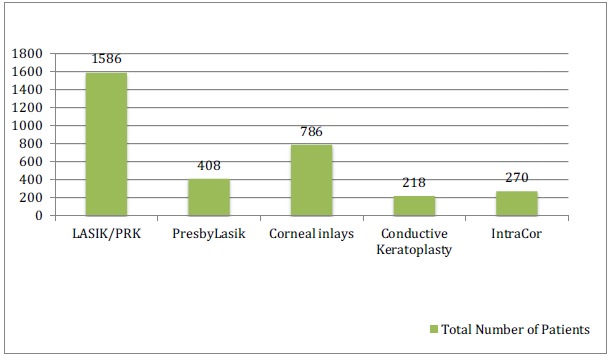
Total Number of Patients Based on Literature Review.

The total numbers of patients in all the studies included in this review article are shown in (Graph **1**). Graph (**2**) shows the post-operative spectacle independence as a percentage of patients. Four studies in the LASIK/PRK group, four studies in the presbyLASIK group, seven studies in the corneal inlays group and two studies in the conductive keratoplasty group reported post-operative spectacle independence. In the LASIK/PRK group, an average of 84% of patients achieved post-operative spectacle independence with a standard deviation of ±13.35 (68%-97% range) and an average follow-up of 12 months. In the presbyLASIK group, 85% ±9.85 (72-91% range) achieved spectacle independence with an average follow-up of 13 months. In the corneal inlay group 48% ±42.2 (2.5-98% range, mean follow-up 16 months) achieved spectacle independence, showing a large amount of variability in the data. In the conductive keratoplasty group, 80% ±3.16 (77-81.5% range) achieved spectacle independence with an average follow-up of 24 months.

**Graph (3):**
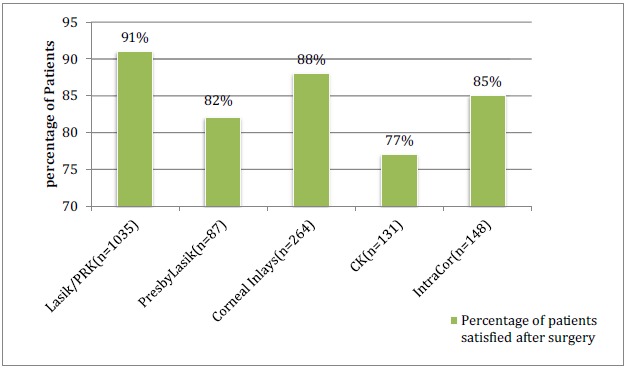
Patient Satisfaction After Surgery (Mean follow-up 14.5 months).

The percentage of patients satisfied with the respective procedures is shown in (Graph **3**). Nine studies in the LASIK/PRK group, four studies in the presbyLASIK group, eight studies in the corneal inlays group, two studies in the conductive keratoplasty group and four studies in the INTRACOR group reported post-operative patient satisfaction. In the LASIK/PRK group, an average of 91% of patients with a standard deviation ±5.2(80-94% range) were satisfied with the procedure and average follow-up was 8 months. In the presbyLASIK group, 82% ±11.58 (76-100% range) were satisfied with the procedure and average follow-up was 7.5 months. In the corneal inlays group, 86% ±7.9 (75-100% range) were satisfied with the procedure and average follow-up was 23 months. In the conductive keratoplasty group, 77% ±3.9 (76-81.5% range) were satisfied post-operatively and average follow-up was 9 months. In the INTRACOR group, 85% ±11 (71-98% range) were satisfied with the procedure and average follow-up was 16 months.

## CONCLUSION

Modifications to existing corneal procedures and recent technological advances have led to the development of creative and efficacious corneal procedures for the treatment of presbyopia. A number of surgical options currently exist for patients who desire independence from spectacles. In determining the appropriate procedure for each individual patient, a detailed discussion should be held between surgeon and patient, with careful assessment of patient age, lifestyle, and occupational needs. Expectations should be clearly determined prior to any surgical intervention. While the above procedures have demonstrated high efficacy and show great promise, we look forward to further advances that aim to reliably re-create the lens’s natural ability to accommodate with good efficacy to reach what has commonly been referred to as the “final frontier” of refractive surgery.

## Figures and Tables

**Fig. (1):**
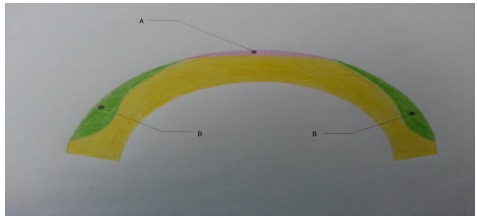
Central/Peripheral PresbyLASIK.

**Graph (2):**
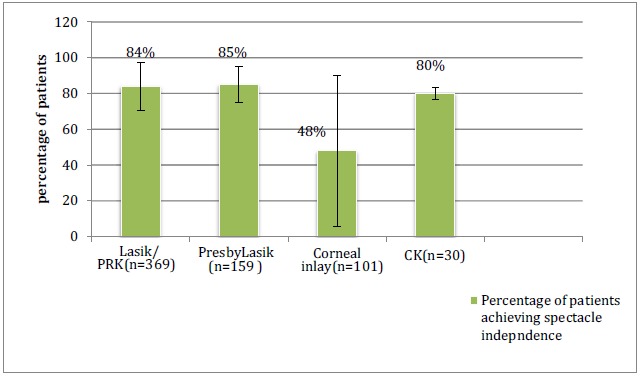
Patients Achieving Spectacle Independence and Standard Deviation (Mean follow-up 16 months).

**Table 1:** LASIK and PRK Monovision Outcomes in Presbyopic Patients. NR = Not Reported.

**Refractive error**	**Author**	**Procedure**	**Number of patients**	**Follow-up in months**	**Postoperative Uncorrected Near Visual Acuity (UNVA)**	**Postoperative Uncorrected Distance Visual Acuity (UDVA)**	**1 Line Loss of Best-Corrected Visual Acuity (% of eyes)**	**2 Lines loss of Best-Corrected Visual Acuity (% of eyes)**	**Post-operative Spectacle Independence** **(% of pts)**	**Post-operative satisfaction** **(% of pts)**	**Re-treatment** **(Number/percentage of patients/eyes)**
Myopia, Astigmatism, Emmetropia, Hyperopia	Falcon *et al.* [53]	LASIK.	173	1-28	98.84% ≤ J3	91.9% ≤ 20/20	NR	0.0058%	97.11%	93.64%	24 patients (13.87%)
Myopia, Astigmatism Emmetropia Hyperopia	Alarcon *et al.* [56]	LASIK.	25	3	More than 90% ≤ J1+	More than 90% ≤ 20/20	NR	NR	NR	92%	NR
Myopia	Levinger *et al.* [54]	LASIK	40	12	94.7% ≤ J1	92.1% ≤ 20/32	0%	0%	90.87%	85.22%	4 patients (10%,) for distance correction.
Myopia	Reilly *et al.* [62]	LASIK	82	6	98.9% ≤ J2	100% ≤ 20/25	NR	NR	NR	97.6%	6 enhancements in near eyes (7%),17 enhancements in distance eyes (21%)
Myopia, Astigmatism	Garcia-Gonzalez *et al.* [57]	LASIK	37	6	91.89% ≤ J3	97.30% ≤ 20/25	NR	NR	NR	NR	0%
Myopia	Reinstein *et al.* [58]	LASIK	136	12.5	96% =J2	99% = 20/20	7%	0.00%	NR	NR	52/272 eyes (19%) 27 eyes for distance. 25 eyes for near.
Myopia	Jain *et al.* [14]	PRK-,PARK or LASIK-PARK	42	7	Mean near vision spherical equivalent of -1.95 diopters.	Mean distance vision spherical equivalent of -0.04 diopters.	NR	NR	NR	88%	NR
Hyperopic astigmatism	Reinstein *et al.* [59]	LASIK	129	12.5	81% =J2	95% =20/20	17%	0.00%	NR	NR	22% eyes of which 50% for near and 50% for distance.
Myopia and Hyperopia	Braun *et al.* [60]	LASIK	172	At least 1 month	93% ≤ J3	87.4% ≤ 20/40	NR	NR	NR	93%	61 patients (77 eyes, 35.5%) had enhancement. 48pts (27.9%) for distance. 17pts (9.9%) for near vision. 12 patients (7%) chose to forego monovision .
Myopia and Hyperopia	Levinger *et al.* [61]	LASIK	114	3	97% ≤ J2	79% ≤ 20/25	NR	NR	92% for distance, 76% for reading .	80%	NR
Myopia and Hyperopia	Miranda *et al.* [63]	LASIK	374	NR	NR	NR	NR	NR	NR	92.5%	NR
Myopia and Hyperopia	Goldberg *et al.* [64]	LASIK	114	6 -28	87.7% ≤ J1	99% ≤ 20/20	NR	NR	70.7% for distance, 68.0% for reading	90.1% at level of 8 or higher (10 = most satisfied)	30/228 eyes (13.2%)
Emmetropia	Reinstein *et al.* [55]	LASIK.	148	12.9	99% ≤ J3	100% ≤ 20/32	12.8%	0%	NR	NR	35/296 eyes (11.8%). 14 eyes for distance. 21 eyes for near.

**Table 2:** PresbyLASIK Outcomes in Presbyopic Patients. NR = Not Reported, CDVA= Corrected distance visual acuity. CNVA= corrected near visual acuity.

**Refractiverror**	**Author**	**Procedure**	**Number of patients**	**Follow-up** **in months**	**Postoperative Uncorrected Near Visual Acuity (UNVA)**	**Postoperative Uncorrected Distance Visual Acuity (UDVA)**	**1 Line Loss of Best-Corrected Visual Acuity (% of eyes)**	**2 Lines loss of Best-Corrected Visual Acuity (% of eyes)**	**Post-operative Spectacle Independence** **(% of patients)**	**Post-operative satisfaction** **(% of patients)**	**Re-treatment** **(Number /percentage of patients/eyes)**
Hyperopia	Abrieu-Lacaille *et al.* [65]	Bilateral central presbyLASIK	29	6	Mean .18 logMAR (approx. J2)	Mean 0.02 logMAR (between 20/20 and 20/16)	NR	NR	86%	79%	NR
Hyperopia	Pinelli *et al.* [67]	Bilateral peripheral presbyLASIK	22	6	Mean 0.84 (between 20/25 and 20/20)	Mean 1.0 (20/20)	4.5% CDVA 4.5% CNVA	NR	NR	100%	6 eyes (12%) for distance vision.
Hyperopia	Alio *et al.* [26]	Bilateral central presbyLASIK	25	6	72% ≤ J3	64% ≤ 20/20	10% CDVA	20% CDVA 52% CNVA	72%	76%	6 eyes (12%) for distance vision.
Myopia, Hyperopia	Luger *et al.* [66]	Bilateral central presbyLASIK	31	12	84% ≤ J1	70% ≤ 20/25	33% lost one line of CDVA. 23% lost one line of CNVA.	3% lost 2 lines of CDVA. 8% lost 2 lines of CNVA.	72%	76%	NR
Myopia, Hyperopia	Epstein *et al.* [25]	Unilateral peripheral presbyLASIK on the non-dominant eye	103	27.4	71.4% ≤ J1+ (hyperopes) 65.3% ≤ J1+ (myopes)	67.9% ≤ 20/20 (hyperopes) 70.7% ≤ 20/20 (myopes)	14.3% hyperopic eyes 10.7% myopic eyes.	NR	91.3%	NR	20/75 myopic eyes (26.6%). 8/28 hyperopic eyes. (28.6%)
	Patel *et al.* [28]	Comparison of 2 IOLs and central presbyLASIK	13	6	Mean UNVA 0.68 (between 20/32 and 20/25)	Mean UDVA 0.80 (20/25)	NR	NR	NR	NR	NR
Hyperopia	Jung *et al.* [27]	Bilateral central presbyLASIK	27	6	64.3% ≤ 0.65 (between 20/32 and 20/25)	64.3% ≤ 20/25	NR	4% CDVA	NR	NR	NR
Myopia, Emmetropia and Hyperopia	Uy *et al.* [24]	Bilateral peripheral presbyLASIK	158	3	83% ≤ J3 (myopes) 87% ≤ J3 (hyperopes or emmetropes)	83% ≤ 20/30 (myopes) 87% ≤ 20/30 (hyperopes or emmetropes)	NR	NR	NR	NR	4 myopic eyes (2.1%). 1 emmetropic eye. (0.8%) 1 hypermetropic eye (0.8%)

**Table 3:** Corneal Inlays Outcomes in Presbyopic Patients. NR = Not Reported.

**Refractive error**	**Author**	**Type of Inlay**	**Number** **of** **patients**	**Follow-up** **in months**	**Postoperative Uncorrected Near Visual Acuity (UNVA)**	**Postoperative Uncorrected Distance Visual Acuity (UDVA)**	**1 Line Loss of Best-Corrected Visual Acuity (% of eyes)**	**2 Lines loss of Best-Corrected Visual Acuity (% of eyes)**	**Post-operative Spectacle Independence** **(% of patients)**	**Post-operative satisfaction** **(% of patients)**	**Re-treatment** **(Number/percentage of eyes/pts.)**
Emmetropia	Limnopoulou *et al.* [41]	Flexivue	47	12	100% ≤ J3	Mean 20/50.	36%	0	93.75%	81.25%	NR
Emmetropia	Garza *et al.* [39]	Raindrop	20	12	100% ≤ 20/32 (approx. J2)	85% ≥ 20/40	NR	0%	84%	95%	1patient explantation.
Hyperopia	Chayet *et al.* [40]	Raindrop inlay + hyperopic LASIK	16	12	100% ≤ 20/32 (approx. J2)	100% ≤ 20/19	NR	0%	NR	100%	1 patient explantation.
Myopia	Garza *et al.* [68]	Raindrop inlay + myopic LASIK.	30	12	100% ≤ 20/32 (approx. J2)	93% ≤ 20/40	NR	0%	98%	90%	0%
Emmetropia	Baily *et al.* [69]	Icolens	52	12	90% N8 or better(approximately J5)	98% ≤ 20/60	77%	NR	2.5%	90%.	11 eyes explantation
Emmetropia	Dexl *et al.* [70]	Kamra inlay	24	12	92% ≤ J3	100% =20/20	16.7%	4.2%	NR	mean score 4.9 *	0%
Emmetropia	Dexl *et al.* [71]	Kamra inlay	32	24	Mean acuity 0.24 logRAD between J2 and J3)	Mean 20/16	3.1%	NR	NR	NR	**0%.**
Patients with phakic IOLs	Huseynova *et al.* [72]	Kamra inlay	3	3	Case 1 J2, Case 2 J4, Case 3 J5	Case 1 20/16, Case 2 20/20, Case 3 20/20	0%	0%	33%	NR	NR
Emmetropia	Seyeddain *et al.* [73]	Kamra inlay	24	24	96% ≤ 20/32 (approx. J2)	100% ≤ 20/32	16.7%	0%??	NR	NR	0%
Emmetropia	Seyeddain *et al.* [74]	Kamra inlay	32	24	96.9% ≤ J3	100% ≤ 20/20	40.6%	9.4%.	12.5% completely independent, 75% reported occasional use.	75%	3 eyes
Emmetropia	Seyeddain *et al.* [75]	Kamra inlay	32	36	97% ≤ J3	100% ≤ 20/32	28.3%	3.1%	12.5% completely independent, 43.7% reported occasional use.	84.5%	3 eyes
Past history of LASIK	Tomita *et al.* [36]	Kamra inlay	223	6	83% ≤ J3	100% ≤ 20/20	14%	0%	Mean score 5.0 **	NR	NR
Hyperopia, Emmetropia Myopia.	Tomita *et al.* [37]	Kamra inlay +bilateral LASIK	180	6	100% ≤ J3 (hyperopes and emmetropes) 95% ≤ J3 (myopes)	100% ≤ 20/40 (hyperopes, emmetropes) 93% ≤ 20/40 (myopes)	NR	CDVA*** 5% of myopes. CNVA**** 2% of myopes.		mean score 4.9 in the hyperopic group, 5.0 in emmetropic and myopic group.**	2 eyes (1.1%)
Emmetropes or post-LASIK	Yilmaz *et al.* [35]	Kamra inlay	39	52.2	96% ≤ J3	97% ≤ 20/32	27% lost more than 5 letters	5%	NR	Generally all patients were satisfied	4 eyes underwent explantation.
Emmetropes	Dexl *et al.* [34]	Kamra inlay	32	60	74.2% ≤ J3	93.5% ≤ 20/32	45.2%	22.6%	NR	83.9%	4 eyes (1/4 explantation).

* on a scale of 1 to 7 (1 = not satisfied, 7 = very satisfied) ** 1 = least satisfied/need reading glasses, 7 = most satisfied/do not need reading glasses *** Corrected distance visual acuity **** Corrected near visual acuity

**Table 4:** Conductive Keratoplasty Outcomes in Presbyopic Patients. NR = Not Reported.

**Study Group**	**Author**	**Number of patients**	**Follow-up** **in months**	**Postoperative Uncorrected Near Visual Acuity (UNVA)**	**Postoperative Uncorrected Distance Visual Acuity (UDVA)**	**1 Line Loss of Best-Corrected Visual Acuity (% of eyes)**	**2 Lines loss of Best-Corrected Visual Acuity (% of eyes)**	**Post-operative Spectacle Independence** **(% of pts.)**	**Post-operative satisfaction** **(% of pts.)**	**Re-treatment** **Number/% of eyes/pts.**
Eyes with no previous surgery and eyes s/p LASIK	Tomita *et al.* [76]	38	12	Mean in the non-LASIK group: 0.71 logMAR (approx. 20/100). Mean in the LASIK group: 0.64 logMAR (between 20/80 and 20/100).	Mean in non-LASIK group: 0.28 logMAR (between 20/30 and 20/40). Mean in the LASIK group: 0.3 logMAR (20/40).	NR	NR	NR	NR	NR
Eyes with binocular monofocal IOL implantation	Ye *et al.* [77]	27	12	0.30 logMAR 100% <_ J5	0.37 logMAR(20/50 or better)	0%	0%	81.48%	81.48%	0%
Near plano	Stahl *et al.* [78]	10	36	78% ≤ J3	78% ≤ 20/20	0%	0%	77%	NR	NR
Hyperopia and Emmetropia	McDonald *et al.* [48]	143	6	85% ≤ J3	85% ≤ 20/25	NR	1%	NR	76%	0%

**Table 5:** INTRACOR Outcomes in Presbyopic Patients. NR = Not Reported.

**Study group**	**Author**	**Number of patients**	**Follow-up**	**Postoperative Uncorrected Near Visual Acuity (UNVA)**	**Postoperative Uncorrected Distance Visual Acuity (UDVA)**	**1 Line Loss of Best-Corrected Visual Acuity (% of eyes)**	**2 Lines loss of Best-Corrected Visual Acuity (% of eyes)**	**Post-operative Spectacle Independence** **(% of pts)**	**Post-operative satisfaction** **(% of pts)**	**Re-treatment** **Number/%of eyes/pts**
Mild Hyperopes	Khoramnia *et al.* [51]	20	36	SDRG* 100% ≤ J1. MDRG** 82.5% ≤ J3. LDRG*** 80.0% ≤ J1.	SRDG: 100%: ≤ 20/32 MRDG: 80% ≤ 20/40. LRDG: 100% ≤ 20/40.	SRDG: 75.0%CDVA, 25.0%CNVA. MRDG: 20.0%CDVA, 40.0% CNVA. LRDG: 33.3%CDVA, 50.0%CNVA.	SRDG: 0.0%CDVA/CNVA. MRDG: 80.0% CDVA, 40.0% CNVA. LRDG: 0.0% CDVA, 16.7% CNVA.	NR	80%	NR
Emmetropes	Thomas *et al.* [79]	20	12	Mean: 20/25 (J1).	Mean: 20/20.	45%	15.0%	NR	83%	NR
Mild hyperopes	Menassa *et al.* [80]	25	18	Median: 0.2 logMAR (approx J2)	Median: 0.201 logMAR	52.0%	26%	NR	NR	NR
Mild hyperopes	Holzer *et al.* [50]	63	12	70.7% ≤ J3	95.0% ≤ 20/40	21.4%	7.1%	NR	71.4%	NR
Emmetropes and mild hyperopes	Bohac *et al.* [81]	72	3	88.23% ≤ J3	mean 20/20	NR	NR	NR	98.0%	NR
Low hyperopes	Holzer *et al.* [82]	25	3	0.26 logMAR (between J3 and J2).	0.05 logMAR (between 20/20 and 20/25)	42%	8%	NR	NR	NR
Low myopia, emmetropia, low hyperopia	Ruiz *et al.* [49]	45	6-12	91.6 J2 or better	89.2% 20/25 or better	0%	0% at 1 year	0%	All patients were generally pleased with their results.	NR

* Small ring diameter group ** Medium ring diameter group *** Large ring diameter group (Graph **1**) Total Number of Patients Based on Literature Review. (Graph **2**) Patients Achieving Spectacle Independence and Standard Deviation (Mean follow-up 16 months) (Graph **3**) Patient Satisfaction After Surgery (Mean follow-up 14.5 months)
